# Improved Photocatalytic and Antioxidant Activity of Olive Fruit Extract-Mediated ZnO Nanoparticles

**DOI:** 10.3390/antiox12061201

**Published:** 2023-06-01

**Authors:** Sadia Ghaffar, Azhar Abbas, Muhammad Naeem-ul-Hassan, Nasir Assad, Muhammad Sher, Sami Ullah, Hassan A. Alhazmi, Asim Najmi, Khalid Zoghebi, Mohammed Al Bratty, Ali Hanbashi, Hafiz A. Makeen, Hatem M. A. Amin

**Affiliations:** 1 Institute of Chemistry, University of Sargodha, Sargodha 40100, Pakistanazhar.ramzan@uos.edu.pk (A.A.);; 2 Department of Chemistry, Government Ambala Muslim Graduate College Sargodha, Sargodha 40100, Pakistan; 3 Department of Pharmaceutical Chemistry and Pharmacognosy, College of Pharmacy, Jazan University, Jazan 82912, Saudi Arabia; 4 Substance Abuse and Toxicology Research Centre, Jazan University, Jazan 82912, Saudi Arabia; 5 Department of Pharmacology, College of Pharmacy, Jazan University, Jazan 82912, Saudi Arabia; 6 Pharmacy Practice Research Unit, Department of Clinical Pharmacy, College of Pharmacy, Jazan University, Jazan 82912, Saudi Arabia; 7 Faculty of Chemistry and Biochemistry, Ruhr University Bochum, 44801 Bochum, Germany; 8 Chemistry Department, Faculty of Science, Cairo University, Giza 12613, Egypt

**Keywords:** zinc oxide, photodegradation, antioxidant activity, methylene blue, methyl orange, green synthesis, photocatalyst

## Abstract

Photodegradation is an efficient strategy for the removal of organic pollutants from wastewater. Due to their distinct properties and extensive applications, semiconductor nanoparticles have emerged as promising photocatalysts. In this work, olive (*Olea Europeae*) fruit extract-based zinc oxide nanoparticles (ZnO@OFE NPs) were successfully biosynthesized using a one-pot sustainable method. The prepared ZnO NPs were systematically characterized using UV-Vis, FTIR, SEM, EDX and XRD and their photocatalytic and antioxidant activity was evaluated. SEM demonstrated the formation of spheroidal nanostructures (57 nm) of ZnO@OFE and the EDX analysis confirmed its composition. FTIR suggested the modification/capping of the NPs with functional groups of phytochemicals from the extract. The sharp XRD reflections revealed the crystalline nature of the pure ZnO NPs with the most stable hexagonal wurtzite phase. The photocatalytic activity of the synthesized catalysts was evaluated by measuring the degradation of methylene blue (MB) and methyl orange (MO) dyes under sunlight irradiation. Improved degradation efficiencies of 75% and 87% were achieved within only 180 min with photodegradation rate constant *k* of 0.008 and 0.013 min^−1^ for MB and MO, respectively. The mechanism of degradation was proposed. Additionally, ZnO@OFE NPs exhibited potent antioxidant activity against DPPH, hydroxyl, peroxide and superoxide radicals. Hence, ZnO@OFE NPs may have potential as a cost-effective and green photocatalyst for wastewater treatment.

## 1. Introduction

Since the industrial revolution, organic pollutants have had serious growing impacts on health and the environment [1]. In particular, the textile dying industry is one of the biggest wastewater polluters and represents a great threat to aquatic life [2]. Hence, it is necessary to develop effective strategies of treatment and/or removal of organic dyes from wastewater to ensure sustainability of the environment for future generations. Among others, photooxidation has emerged as an effective approach for decoloration and degradation of dyes and wastewater remediation as it is more efficient, less expensive and environmentally benign compared to traditional methods of water treatment such as biological processes, flocculation and chemical treatment [3,4,5]. Since the pioneering work by Fujishima and Honda in 1972 on photocatalytic water splitting [6], the field of photocatalytic degradation of pollutants has widely expanded. In this context, semiconductor photocatalysis has emerged as a promising method to degrade various pollutants at ambient temperature and pressure using nanoparticles. It is therefore necessary to develop highly efficient photocatalytic materials for oxidative degradation.

Several semiconductors such as TiO_2_, ZnO, WO_3_ and ZrO_2_ have been widely studied as photocatalysts for decomposition of various dyes [7,8,9]. Among these, the application of ZnO and TiO_2_ NPs has significantly expanded in photocatalytic systems, solar cells and light-emitting devices [10,11,12]. However, the use of ZnO is economically more favorable at a large scale than TiO_2_ as it is cost-effective [13]. As a photocatalyst, ZnO has attracted great interest due to its desired characteristics including nontoxicity, wide band gap, high binding energy, photosensitivity and outstanding chemical stability at room temperature [8,14]. In addition, numerous studies have demonstrated the potential of ZnO as antibacterial and antioxidant agent [15,16]. All of these intrinsic and simply tunable properties are advantageous to its use in diverse domains such as wastewater treatment, biomedicine and optoelectronic devices [17,18].

ZnO NPs have been synthesized using a wide range of physical and chemical routes [19,20,21]. However, these physicochemical methods, though some are technically viable, are accompanied with the use of hazardous chemicals, high-energy, long-time consumption and high cost. Alternatively, green synthesis of NPs has gained wide interest because of its low cost, low toxicity and eco-friendly nature [22,23]. Using plant extracts offers a green and sustainable opportunity for exploring the biosynthesis of ZnO NPs and is both more environmentally benign and more rapidly progressive than conventional chemical methods [16,24]. Numerous plant extracts such as *Lamiaceae* [25], *Ziziphus jujube* [26], *Lepidium sativum* [27] and *Azadirachta indica* [28] and, in addition, numerous bioactive compounds in plant extracts (such as polyphenols, ketones, flavonoids, carboxylic acids, proteins and amides) have been recognized as acting as reducing and stabilizing agents [29,30]. For example, a biosynthesized ZnO NPs using *Solanum nigrum* extract exhibited higher degradation efficiency for methylene blue dye than the chemically synthesized ZnO NPs [31]. Mutukwa et al. recently reviewed the application of *Lamiaceae*-derived ZnO NPs in the photodegradation of organic dyes and as antibacterial agents [25]. In a previous report, ZnO NPs (41 nm) were synthesized based on the leaf extract of *O. europaea*; however, no application was reported in this study [22].

In this work, we report on the facile and green synthesis of ZnO NPs using olive fruit (*Olea europaea*) extract and explore their photocatalytic degradation of methylene blue (MB) and methyl orange (MO), as model dye molecules, under diffused sunlight irradiation. The use of natural solar light can significantly reduce the cost of the photocatalytic degradation. The successful synthesis of ZnO@OFE NPs was confirmed using FTIR and UV-Vis spectroscopic techniques. The biosynthesized ZnO@OFE NPs were further analyzed using scanning electron microscopy (SEM) along with energy-dispersive X-ray (EDX) and X-ray diffraction (XRD) to determine their morphology, composition and structure. The as-synthesized ZnO@OFE NPs revealed high degradation efficiency toward both dyes. Additionally, the ZnO@OFE NPs exhibited promising antioxidant activity against DPPH radical, hydroxyl, hydrogen peroxide and superoxide radicals. The results render our approach feasible, sustainable and cost-effective for the photodegradation of dyes and antioxidant applications in wastewater.

## 2. Materials and Methods

### 2.1. Materials

Fresh olive fruits (*O. europaea*) were identified and harvested by the Barani Agriculture Research Institute (BARI), Chakwal, Punjab, Pakistan, during the month of October. Olive trees were 8–10 years old, and the fruits used were ripe, weighed on average 3 g and had a size of 2–3 cm. The quality of the collected samples was identified by the Department of Botany, University of Sargodha, Pakistan. ZnCl_2_۰7H_2_O, methylene blue (MB) and methyl orange (MO) were of analytical grade and used without further purification. Distilled water was used to prepare all solutions.

### 2.2. Preparation of the Olive Fruit Extract (OFE)

Ethanolic extract of *O. europaea* fruit was prepared using a maceration method. In the typical procedure, olive fruits were thoroughly washed with distilled water to remove dust and impurities. The fruit pulp was separated from the stone, shade dried and then grinded into powder. Ten grams of olive fruit powder was added into 100 mL ethanol. This solution was macerated and stirred for 8 h on magnetic stirrer at room temperature and afterwards was filtered through Whatman filter paper. The filtrate was then put into water bath until a semi-solid form of the extract was obtained and stored in Eppendorf tubes for further experiments.

### 2.3. Biosynthesis of O. europaea-Mediated ZnO NPs

ZnO NPs were prepared following a reported procedure by Osuntokun et al., with slight modification [32]. Briefly, 0.68 g of ZnCl_2_.7H_2_O was dissolved in 100 mL of water in a glass container. Afterwards, 20 mL of the *O. europaea* extract suspension was added to the Zn^2+^ solution and was stirred for 30 min. The mixture was then heated under stirring on a hot plate at 70 °C for 20 min until a brownish precipitate was observed, which manifested the completion of the reaction. The product was washed with ethanol and water. Finally, the product was transferred to Petri dishes, and calcined in an oven at 450 °C, yielding ZnO. This sample was collected and powdered to obtain the herein called “ZnO@OFE” NPs.

### 2.4. Characterization of ZnO@OFE

The as-synthesized ZnO NPs were characterized using a UV-Vis spectrophotometer (UV-1700 Pharmaspec Shimadzu, Kyoto, Japan) in the range of 800–300 nm. To identify the functional groups of possible molecules of the extract on the particle surface, Fourier transform infrared (FTIR) spectra of the ethanolic OFE extract and the prepared ZnO@OFE NPs were collected using Shimadzu FTIR 8400 Spectrometer in the region of 4000–350 cm^−1^ with the KBr pellets method. The crystalline structure of ZnO@OFE NPs was analyzed using X-ray diffraction XRD (JDX-3532, JEOL, Tokyo, Japan,) with CuKα = 1.54056 Å over a 2*θ* range of 10–80° at accelerating voltage of 40 kV and current strength of 15 mA. The particles’ morphology and elemental composition of ZnO@OFE NPs were investigated using scanning electron microscopy (SEM, Nova NanoSEM NPE-218) at an operating voltage of 10 kV and the energy-dispersive X-ray (EDX, JEOL EDX system) was recorded at an operating voltage of 20 kV.

### 2.5. Adsorptive Study of ZnO@OFE

Prior to investigating the photocatalytic activity of the as-synthesized particles, the adsorptive capacity of the studied dyes onto the synthesized particles was identified in the absence of illumination. Batch experiments were conducted in a typical room environment without illumination to explore whether the ZnO@OFE NPs would absorb the dyes (MB and MO). Briefly, 60 mL of both dyes MB and MO (10 ppm) were placed in conical flasks with and without ZnO@OFE NP adsorbent and kept on orbital shaker for 60 min. After filtration using Whatman paper, UV-Vis spectrophotometer was used to monitor the progress of reaction by measuring absorbance at intervals of time. The absorbance decreased over time, indicating that the ZnO@OFE NPs were capable of adsorbing both dyes.

### 2.6. Photocatalytic Activity of ZnO@OFE NPs

The photocatalytic activity of the NPs was evaluated through the decolorization of MB and MO in aqueous solution under direct diffused sunlight irradiation. The photocatalytic activity experiments were conducted under direct solar light exposure (i.e., full spectrum light from UV to IR, UV index between 7 and 10 as measured using a mobile app) on clear sunny days between 10 am and 2 pm. First, 30 mL of both dyes’ solutions (10 ppm) with various amounts of ZnO@OFE NPs (5, 10, 15, 20, 25 and 30 mg) was agitated in the dark on an orbital shaker for 30 min to find out the maximum absorption of MB and MO on the photocatalyst’s surface. It was found that catalyst dose of 30 mg in 30 mL provides good absorption and was used in the detailed photoactivity study. Afterwards, the solutions were exposed to sunlight irradiation under stirring for three hours. Finally, 3 mL from each solution was collected after 0, 30, 60, 90, 120 and 180 min of sunlight exposure, which was then centrifuged to remove the catalyst particles and the residual dye solution was quantified by measuring the absorption spectra using UV-Vis spectrophotometer. For the UV measurements, triplicates were carried out and a difference of maximum 5% between individual measurements was obtained. The characteristic absorbance peaks of MB at 665 nm and for MO at 460 nm were used as a measure of the concentration of the dyes in solution.

### 2.7. Antioxidant Activity

#### 2.7.1. 2,2-Diphenyl-1-picrylhydrazyl (DPPH) Radical Scavenging Assay

The ability of NPs to donate hydrogen or scavenge radicals was measured by comparing their free radical scavenging activities to those of the standard gallic acid. Adapting the method of Blois [33], 1 mL of variable concentrations of the ZnO NPs@OFE or the standard (12.5, 25, 50, 100 and 200 g mL^−1^) was added to 3 mL of DPPH radical solution (0.1 mM in ethanol). The solution was kept in the dark for 30 min by folding aluminum foil over it. The decrease in solution absorbance was then measured at 517 nm.

#### 2.7.2. Hydroxyl Radical Scavenging Assay

The hydroxyl radical scavenging activity was measured based on a modified method by Kunchandy and Rao [34]. Hydroxyl radicals are generated using the Fe^3+^ ascorbate-EDTA-hydroperoxide system (Fenton reaction). The reaction mixture consists of 2.8 mM 2-deoxy-2-ribose, 20 mM KH_2_PO_4_-KOH buffer (pH 7.4), 0.1 M FeCl_3_, 0.1 M EDTA, 1.0 mM H_2_O_2_, 0.1 M ascorbic acid and different quantities (0–200 g mL^−1^) of ZnO@OFE NPs and gallic acid was used as a reference material. After an hour of incubation of the mixture at 37 °C, 1 mL of 2.8% trichloroacetic acid was added to 0.5 mL of the reaction mixture, followed by the addition of 1 mL of 1% aqueous thiobarbituric acid, and thereafter the resultant liquid was heated for 15 min at 90 °C and the solution was colored. After cooling, absorbance was measured at 532 nm against a blank solution in a UV-Vis spectrophotometer.

#### 2.7.3. Hydrogen Peroxide Scavenging Activity

The peroxide scavenging experiments were carried out with minimal adjustments to the method of Cetinkaya et al. [35]. Briefly, 2 mL of hydrogen peroxide solution (100 mM) was added to 1 mL of ZnO@OFE NPs suspension (in phosphate buffer, pH 7.4) containing various concentrations of the samples (12.5, 25, 50, 100 and 200 µg mL^−1^ of ZnO NPs@OFE and gallic acid). After 10 min of room-temperature incubation, the absorbance of hydrogen peroxide at 230 nm was measured and compared to that of a phosphate buffer solution without hydrogen peroxide taken as a blank. The absorbance at 230 nm of a reference sample of hydrogen peroxide also solution served as a useful control.

#### 2.7.4. Superoxide Radical Scavenging Assay

In order to measure the superoxide (O_2_^•−^) radical scavenging capacity, a previous nitro blue tetrazolium (NBT) reduction assay was followed with minor changes [36,37]. The reaction mixture contained 1 mL of NBT (1.0 M in 100 mM PBS, pH 7.4), 0.1 mL of 50 mM phosphate buffer solution (PBS, pH 7.4) and 1 mL of NADH solution (1.0 M in 100 mM PBS, pH 7.4) and 1 mL of the various concentrations (12.5, 25, 50, 100 and 200 g mL^−1^) of samples (ZnO@OFE NPs and gallic acid). The reaction was started by adding 100 µL of phenazine methosulfate solutions (60 µM) and thereafter the reaction mixture was incubated at 37 °C for 1 h. Using a UV-Vis spectrophotometer, the absorbance of the solution was measured at 530 nm against the corresponding blank solution.

## 3. Results and Discussion

### 3.1. Green Synthesis and Characterization of OFE-Mediated ZnO NPs (ZnO@OFE)

The biosynthesis of ZnO@OFE NPs was performed using a simple two-step method, following a recently published protocol [32]: first, Zn(OH)_2_ was synthesized from ZnCl_2_, and then this intermediate product was calcined into ZnO NPs by heating at 450 °C. With the help of phytochemicals in the extract such as polyphenols and flavonoids, a possible mechanism involves the Zn ions being separated from the solvating anionic counterparts and then being reduced to the more stable metallic Zn by chelating to the phytochemicals. When the OH group of the phytochemicals binds to Zn^2+^, Zn(OH)_2_ was formed as a white milky precipitate. To obtain ZnO NPs, this intermediate product was dried in an air-drying oven at 70 °C for 6 h, and afterwards calcined at 450 °C. We noted that the polyphenols in in O. euorpeae contain many OH^−^ groups that are possibly involved in the conversion of ZnCl_2_ to Zn(OH)_2_ through weak hydrogen bonds between polyphenols and metal chloride. To confirm the successful synthesis of OFE-functionalized ZnO NPs, the sample was characterized using various spectroscopic and microscopic techniques. the photocatalytic activity of the synthesized samples is discussed later in this article.

#### 3.1.1. Powder X-ray Diffraction (XRD) Characterization

**Figure 1:**
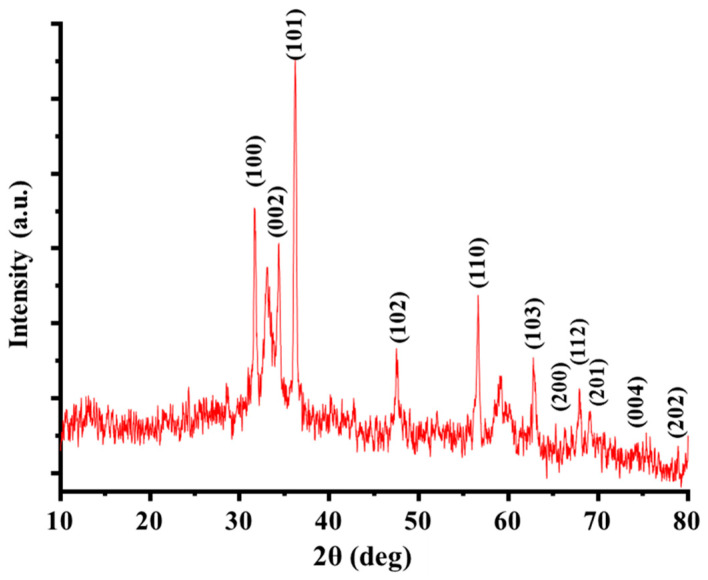
Powder XRD pattern of ZnO@OFE NPs.

The crystalline structure and phase purity of ZnO@OFE NPs was confirmed by XRD measurement. Figure 1 shows the XRD pattern of ZnO NPs synthesized by *O. europeae*. The spectrum featured all diffraction peaks that correspond to the hexagonal wurtzite phase of ZnO NPs. Bragg reflections with 2*θ* values of 31.7°, 34.3°, 36.2°, 47.5°, 56.6°, 62.7°, 67.9° and 69° were observed corresponding to (100), (002), (101), (102), (110), (103), (200), (112), (201), (004) and (202) planes of the wurtzite phase (JCPDS Card No. 36-1451), respectively. Similar patterns were also reported for ZnO NPs [22,32]. The observed peaks are sharp, suggesting the high crystalline nature. Apart from the two small peaks observed at 2*θ* values of 31° and 59°, which might be related to the extract, no other typical peaks of foreign phases were observed, approving the formation of phase-pure ZnO. The crystallite or grain size (*D*) of the as-synthesized ZnO NPs was calculated from the XRD data using Scherrer’s equation as follows:

(1) D=Kλβcos θ

where *k* = 0.9 is the Scherrer constant, *λ* = 0.15406 nm represent the wavelength of the uses X-ray source, *β* = FWHM is the full width at half maximum of the peaks in radians and *θ* is the peak position in radians. For the studied particles, an average crystallite size of 24.3 ± 3.3 nm was obtained. The crystallite site is roughly half the particle size obtained from SEM (56.8 nm, see below), suggesting that the particles compromise a few smaller grains.

#### 3.1.2. FTIR Analysis

**Figure 2:**
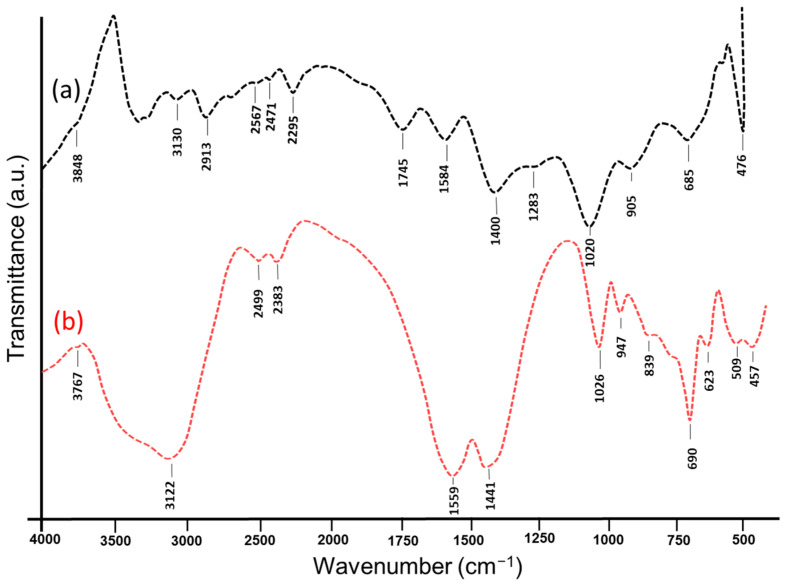
FTIR spectra of (**a**) *O. europeae* fruit extract OFE and (**b**) synthesized ZnO NPs@OFE.

The dual action of the phytochemicals in the plant extract in the reduction of Zn ions and the stabilization of ZnO@OFE NPs were studied using FTIR spectroscopy. Figure 2 compares the spectra of the synthesized ZnO NPs and the sole OFE extract. The absorption peaks of both OFE and ZnO@OFE NPs were used to confirm the functionalization of the particles. The FTIR peaks of the formed ZnO NPs are similar to that of the fruit extract, evidencing that the compounds, mainly phytochemicals, present in the OFE act not only as a reducing agent of silver ions, but also as a capping agent of the synthesized particles. The spectra for ZnO@OFE revealed the presence of various absorption peaks at 3767, 3122, 2499, 2383, 1599, 1441, 1026, 947, 839, 690, 623, 509 and 457 cm^−1^. The wide intense band at 3122 cm^−1^ can be assigned to O–H stretching of polyphenols in the extract and possibly with some N–H stretching of amine overlapping [38]. This band for ZnO NPs is shifted lower wavelength than that of OFE, indicating the binding of Zn^2+^ with these hydroxyl or amine groups [39]. The peak at 1559 cm^−1^ can be attributed to amide I vibrations of proteins and was shifted to 1559 cm^−1^ in the ZnO NPs due to linking of the proteins with the surface of the NPs [39]. The tertiary alcohol (C-OH) group contributes with a peak at 1441 cm^−1^. Since carboxylic acids absorb radiation usually around 1026 cm^−1^, their C–O stretching vibrations create a narrow absorption band, which was also observed at similar value (1020 cm^−1^) for the extract. Additionally, two new peaks appeared at 457 and 623 cm^−1^ in the IR spectrum of ZnO@OFE NPs, which are characteristic of bending vibrations of Zn–O bonds, in agreement with previous reports [40]. The FTIR results of ZnO NPs and *O. europeae* fruit extract conclude a significant modification of the surface of the NPs with phytochemicals, mainly phenolic compounds as suggested by Ghanbari et al., which plays a role in the formation and stabilization of the ZnO NPs [41].

#### 3.1.3. UV-Vis Analysis of ZnO@OFE NPs

**Figure 3:**
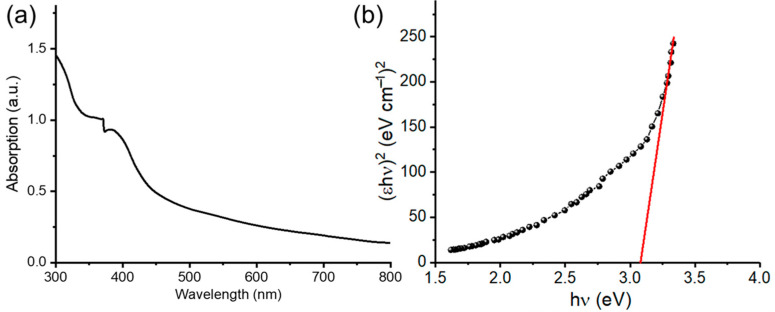
(**a**) UV-Vis absorption spectrum of ZnO@OFE NPs. (**b**) Tauc plots for the determination of optical band gap of our semiconductor material.

UV-Vis spectroscopy was first used to assess the optical properties of the fabricated NPs. Figure 3a displays the absorption spectrum of ZnO@OFE NPs in suspension. The spectrum revealed the characteristic peak at about 382 nm, confirming the formation of ZnO@OFE in the nanoscale. The appearance of such a peak is related to the nanoparticles’ localized surface plasmon resonance (LSPR) property, which results from the oscillations of the electron cloud surrounding the nanoparticles when they were aligned in resonance with the wavelength of irradiation light. This peak showed a red shift (17 nm) compared to bulk ZnO (365 nm), which reflects the nanosize and the quantum confinement effects, which causes the optical properties of semiconductor NPs to differ from those of their bulk counterparts [42]. Similar results were reported by Pudukudy et al. [20] The optical band gap (*E_g_*) of the synthesized ZnO particles from the UV data using Tauc’s equation: the following equation: (*εhν*)^2^ *= K*(*hν − E_g_*) where *h* is the Planck’s constant, *ν* is frequency, *ε* is molar extinction coefficient, *K* is energy independent constant and *n* depends on the type of transition and *n* = 2, for direct allowed band gap materials. The average band gap was estimated from the intercept of linear portion of the (*εhν*)^2^ vs. *hν* plot on the *x*-axis as shown by the red line in Figure 3b. *E_g_* of 3.09 eV was obtained for ZnO@OFE NPs, which is lower than that of bulk ZnO (3.37 eV).

#### 3.1.4. SEM-EDX Characterization

**Figure 4:**
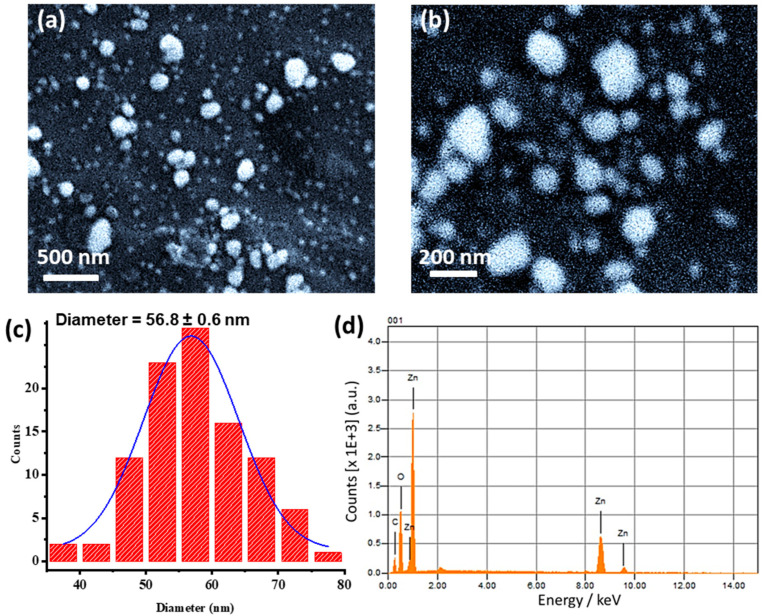
SEM images of the synthesized ZnO NPs with low (**a**) and high (**b**) magnification; (**c**) corresponding particle size distribution; (**d**) EDX spectra of OFE@ZnO NPs.

The morphology and particle size were examined using SEM. SEM images of the particles were recorded with two magnifications as shown in Figure 4a,b. The images demonstrated almost spheroidal particles with an average particle size of 56.8 ± 0.6 nm. The image showed individual particles as well as a number of aggregates, which might also have occurred during drying for SEM measurements. The particle size analysis (Figure 4c) was performed using ImageJ software. The spherical form of ZnO@OFE NPs is similar to in previous studies [20,22]. The efficiency of NPs against infections is highly dependent on their shape. Since spherical NPs are more effective against bacteria as they can easily penetrate into the cell wall of pathogens, ZnO@OFE NPs would have a promising role in fighting clinical pathogens [43]. In previous reports, ZnO NPs were synthesized using *Cassia fistula* [44] or *Pongamia pinnata* [45] extract and the particles revealed larger aggregates (~100 nm) rather than individual NPs. Moreover, smaller capping agents such as citrate showed the formation of particles in the µm size [46], pointing out to the effective role of OFE extract in stabilizing and capping the synthesized NPs.

In order to determine the elemental composition of the synthesized ZnO@OFE NPs, EDX analysis was performed as shown in Figure 4d. This analysis verified the presence of only zinc and oxygen elements present in ZnO@OFE NPs without any other elemental contaminations.

### 3.2. Adsorptive Assay of MB and MO Dyes

**Figure 5:**
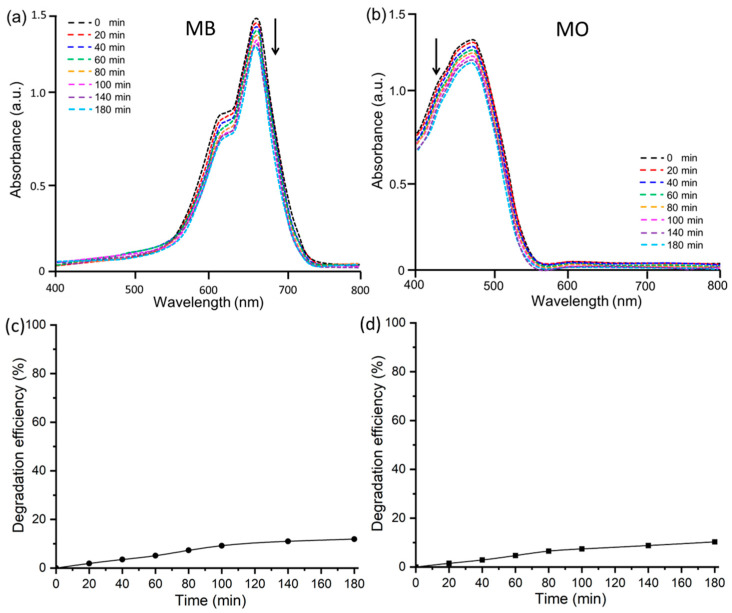
UV–Vis absorption spectra at different times for the adsorptive kinetics of (**a**) MB and (**b**) MO Dyes by ZnO@OFE NPs without direct sunlight exposure. The corresponding degradation efficiency for dyes (**c**) MB and (**d**) MO.

To investigate first the removal of the dyes via the adsorption pathway, UV-Vis spectra of MO and MB dyes in ZnO@OFE NPs suspension without any irradiation were recorded at different time intervals (0, 20, 40, 60, 80, 100, 140 and 180 min), as shown in Figure 5a,b. The removal/degradation efficiency in % was then calculated using the following equation:

(2) $$\%\mathrm{degradation}=\frac{A_{0}-A_{t}}{A_{0}}\times 100=\frac{C_{0}-C_{t}}{C_{0}}\times 100$$

where *A*_0_ and *A_t_* are the initial absorbance of the dye solution at *t* = 0 min and the absorbance after a removal/degradation time *t,* respectively; whilst *C*_0_ and *C_t_* represent the initial dye concentration and the concentration after time *t*, respectively. As shown in Figure 5a,b, the absorbance only slightly decreased over time for both days, and accordingly the removal efficiency marginally increased with time (Figure 5c,d).

In the absence of irradiation, the removal efficiency by adsorption on the surface of ZnO@OFE NPs reached only 12% for MB dye and 10% for MO dye after 180 min. Similar removal percentages (~12% within 60 min) were reported for MB and MO at ZnO NPs [47]. To explore whether the photoactive ZnO@OFE NPs have any effect on dye removal by photodegradation, similar measurements under direct sunlight exposure were conducted.

### 3.3. Photocatalytic Activity of ZnO@OFE NPs against MB and MO Dyes

In this section we evaluate the potential of the greenly synthesized ZnO@OFE NPs as efficient photocatalyst that, when exposed to UV light from the sunlight, breaks down potentially harmful organic pigments in aqueous media.

#### 3.3.1. Photocatalytic Degradation of Methylene Blue

**Figure 6:**
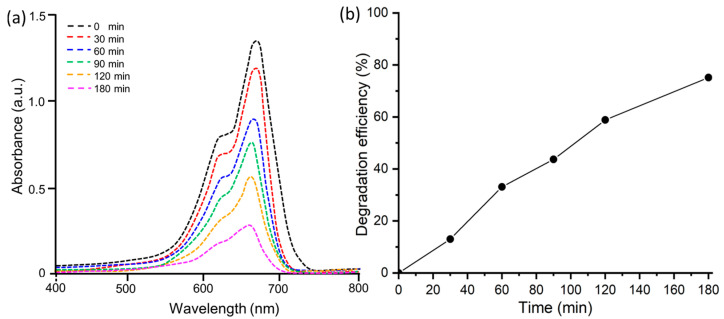
Photocatalytic degradation of MB dye (**a**) UV-Vis spectra at various time intervals; (**b**) degradation efficiency of MB by ZnO@OFE NPs in the presence of direct sunlight.

The photocatalytic degradation activity of MB (cationic dye) by ZnO@OE NPs was examined under sunlight irradiation. Visually, the deep blue color of MB turned into light blue over time under continuous exposure of sunlight. The degradation of the dye was monitored quantitatively by recording its UV-Vis spectra at different time intervals (0, 30, 60, 90, 120 and 180 min), as shown in Figure 6a. From the recorded spectra, it is clear that the intensity of the characteristic peak of MB at λ_max_ of 663 nm decreases with time, showing the decay of the dye concentration and in turn its degradation. Figure 6b displays a plot of the percentage of degradation versus time. This figure reveals that the percentage of degradation increases gradually with time and 75% of MB degraded at ZnO@OFE catalyst within 3 h under sunlight illumination. In addition, no new peaks for intermediate products appeared in the UV-Vis. This photocatalytic effect is significant compared to when the reaction was carried out for the period in the presence of ZnO@OFE without light irradiation (only ~12%). Thus, about 65% more of MB was removed during the same time under UV light irradiation. Comparable activities were previously reported for MB at ZnO [48].

#### 3.3.2. Photocatalytic Degradation of Methyl Orange

**Figure 7:**
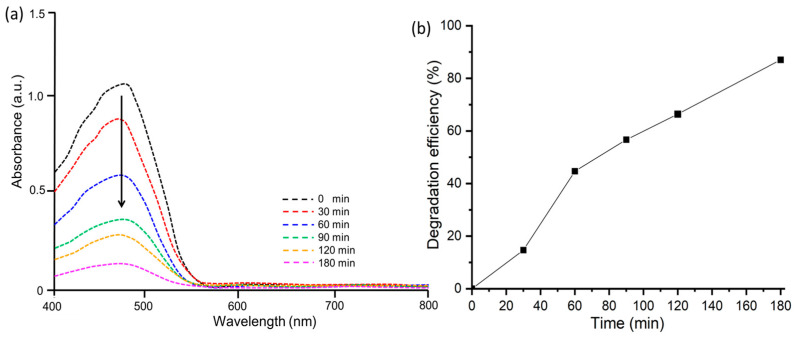
Photocatalytic degradation of MO dye (**a**) UV-Vis spectra showing degradation of MO with time. (**b**) Removal efficiency of MO by ZnO@OFE NPs in the presence of sunlight.

Furthermore, the photocatalytic degradation activity of MO (anionic dye) by ZnO@OFE NPs was conducted in the presence of sunlight using the same procedure as for MB dye. A color change of dye under continuous exposure of sunlight was observable. Figure 7a shows the UV-Vis spectra at various time intervals for MO at ZnO@OFE NPs under illumination. The characteristic peak of MO at λ_max_ of 464 nm was observed and its intensity decreased with time, showing the removal of the dye. Accordingly, the MO degradation increased with time, peaking up to 87% within 180 min of irradiation (Figure 7b), which is in agreement with previous activities reported for ZnO [47]. Although the MO dye is adsorbed on the surface of ZnO NPs by about 10% in the absence of irradiation, the same dye photodegraded under irradiation manifolds (i.e., 77% higher) within the same period. A summary of the photodegradation properties for both MB and MO dyes at ZnO@OFE particles is provided in Table 1.

#### 3.3.3. Kinetics Rate of Photodegradation of MO and MB at ZnO NPs

**Figure 8:**
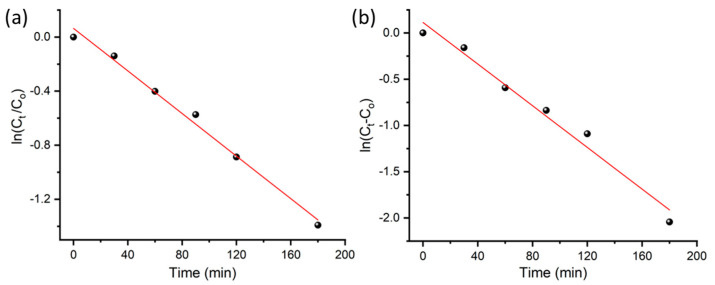
Kinetic study: plot of *ln*(*C_t_/C_o_*) vs. time for the first-order photocatalytic degradation of (**a**) MB and (**b**) MO at ZnO@OFE NPs under sunlight irradiation.

To study the kinetics of this photocatalytic reaction, a plot of *ln*(*C_t_*/*C*_0_) versus irradiation time was established (Figure 8), and a linear relationship was obtained based on the following equation:

(3) $$\ln\left(C_{t}/C_{0}\right)=-kt$$

where *C_t_* denotes the concentration at time interval *t*, *C*_0_ represents the concentration at zero reaction time and *k* is the rate constant, which is obtained using the slope of the straight line. For both dyes, a reasonable linearity was obtained, revealing first-order reaction kinetics for the photodegradation of both MO and MB. The rate constant *k* obtained for MB is 0.0079 min^−1^ with a correlation coefficient *R^2^* of 0.991, whilst that for MO is 0.0113 min^−1^ with *R*^2^ of 0.978. Under the same conditions, the degradation rate of MO is higher than MB (0.008 min^−1^). Comparable kinetic rate constants were previously obtained at ZnO catalysts as summarized in Table 2. Table 2 also compares the photocatalytic performance of our catalyst and ZnO prepared by other plant extracts as well as the P25 TiO_2_ commercial catalyst from the literature.

#### 3.3.4. Mechanism of Photodegradation at ZnO

One possible issue with the use of dyes as model molecules for assessing the photocatalytic activity of semiconductors is that degradation intermediates might absorb at the same wavelength of the dye, causing interference [55]. Furthermore, for sensitized dyes, the degradation could be originated from either an actual photocatalytic process or a dye sensitization effect (i.e., the dye itself absorbs the radiation not the semiconductor) or both [56]. Hence, it is important to distinguish between both effects to assess the effectiveness of the synthesized catalyst and obtain insights into the degradation mechanism. Phenol was recommended as a reasonable molecule to differentiate between both effects for irradiations above 300 nm [56]. Hence, photodegradation experiments were carried out for 4-nitrophenol (4-NP) at ZnO@OFE under the same conditions of the studied dye.

**Figure 9:**
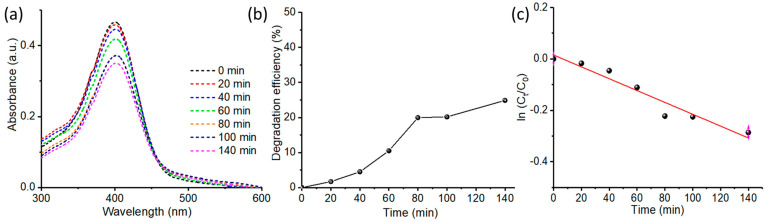
Photocatalytic degradation of 4-nitrophenol in the presence of direct sunlight (**a**) UV-Vis spectra at various time intervals, (**b**) degradation efficiency at ZnO@OFE NPs and (**c**) plot of *ln*(*C_t_*/*C*_0_) vs. time.

Figure 9a displays the UV-Vis spectra of 4-NP solution containing ZnO@OFE NPs. An absorption peak centered at 400 nm was observed and its intensity decreases with time, inferring photodegradation. This result suggests an actual semiconductor mechanism rather than a photosynthesized process and thus supports the effective photocatalytic activity of the synthesized catalyst. As shown in Figure 9b, the degradation efficiency increased slowly with sunlight irradiation time, reaching 25% after 140 min. We note that when the dye solution was left for a few hours, the degradation efficiency reached 77%. The kinetics of degradation was also determined as shown in Figure 9c. The plot in Figure 9c provided a reasonable straight line, indicating first-order kinetics for 4-NP. A rate constant for 4-NP of *k* = 0.002 min^−1^ with *R*^2^ of 0.938 was obtained. Similar photocatalytic rate constants (*k* = 0.002 – 0.003 min^−1^) were reported for 4-NP at biologically synthesized ZnO particles [57].

**Figure 10:**
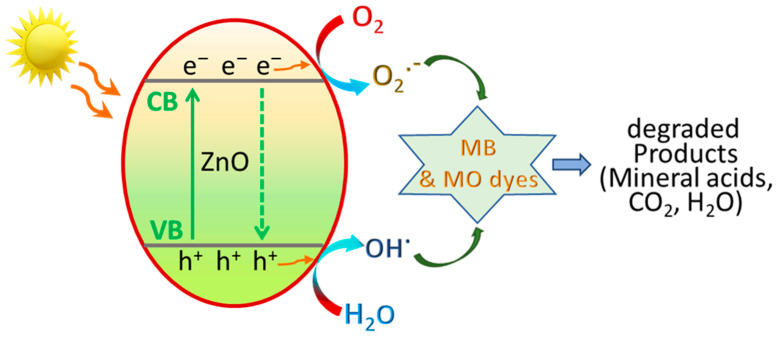
Schematic illustration of the possible photocatalytic degradation of MB and MO at ZnO@OFE NPs.

According to the literature and our results, [58,59] the mechanism of photocatalytic degradation of MB and MO at ZnO NPs can be illustrated as in Figure 10. In general, the photocatalytic degradation mechanism at ZnO involves the following steps [58,59]:
Generation of electron–hole pairs:ZnO + hν → ZnO + e^−^ + h^+^Water photooxidation:h^+^ + H_2_O → OH^•^ + H^+^Oxygen photoreduction:O_2_ + e^−^ → O_2_^•−^Dyes degradation:OH^•^ + O_2_^•−^ + organic dye (MB/MO) → mineral acids + CO_2_ + H_2_O

Sunlight irradiation of the semiconductor ZnO generates electron (e^−^) and hole (h^+^) pairs, that are involved in the photogeneration of radicals. At the surface of ZnO NPs, water is photo-oxidized by the holes (h^+^) to hydroxyl radicals (OH^•^), whilst dissolved oxygen in solution is photo-reduced to superoxide radical anion, as illustrated in the above scheme. The produced active oxygen species and hydroxyl free radicals then oxidize the dye molecules, causing degradation of dyes into less harmful products, mineral acids, CO_2_ and H_2_O [59].

### 3.4. Antioxidant Activity

The scavenging percentage of all studied free radicals was calculated according to the following equation:

(4) $$Scavenging\left(\%\right)=\frac{A_{c}-A_{s}}{A_{c}}\times 100$$

where *A_c_* and *A_s_* are the absorbance for the control (gallic acid + free radical solution) and the sample (ZnO@OFE + free radical solution), respectively. An important metric for antioxidant capacity is the IC_50_ value, which is the half maximum inhibitory concentration. This value represents the concentration of the sample that can scavenge 50% of the free radical. The lower the IC_50_ value, the less amount of antioxidant is required to scavenge the sample, and thus the higher is the antioxidant capacity. IC_50_ is obtained from the linear regression of the plot of scavenging % versus the concentration, by placing the Y-value as 50 and calculating the X-variable in the regression equation.

#### 3.4.1. DPPH Scavenging Assay

**Figure 11:**
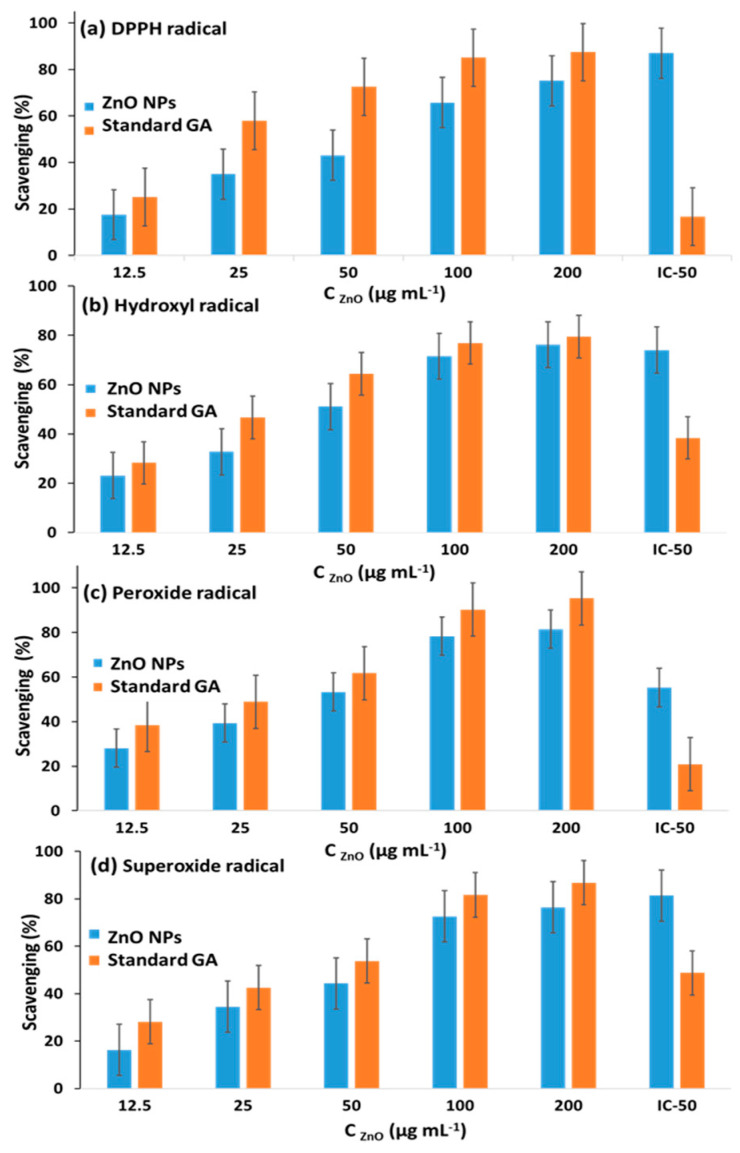
Antioxidant scavenging activity at various concentrations of ZnO@OFE NPs and the standard gallic acid towards (**a**) DPPH radical, (**b**) hydroxyl radical, (**c**) hydrogen peroxide radical and (**d**) superoxide radical. The measurements were carried out in triplicate and the data show mean value ± standard deviation.

Phytochemicals from plant extracts were reported as promising antioxidants [60]. Therefore, we studied and compared the scavenging performance of the synthesized ZnO@OFE NPs with standard gallic acid. The reaction between the antioxidant NPs and the instable free radical DPPH^•^ results in the production of the stable 1,1-diphenyl-2-picryl hydrazyl [47]. Thus, at an absorbance of 517 nm, the capacity to scavenge the free radical DPPH was evaluated. Figure 11a shows DPPH radical scavenging assay and its percentage inhibition increases with increase in the concentration of ZnO@OFE NPs. A significant scavenging (~75%) was obtained at 200 µg mL^−1^, which just a bit less than gallic acid. The scavenging activity data showed an IC_50_ value of 87.04 µg mL^−1^ (*R*^2^ = 0.842). Gallic acid was taken as a standard material and showed IC_50_ value of 16.65 µg mL^−1^ (*R*^2^ = 0.587). This antioxidant activity against DPPH^•^ is better or at least comparable to that (~70%) previously reported for *Curcuma longa*-mediated ZnO particles with the same concentration [61].

#### 3.4.2. Hydroxyl Free Radical Scavenging Assay

The OH free radical scavenging test results are shown in Figure 11b. This assay shows that both ZnO@OFE NPs and standard gallic acid were able to inhibit hydroxyl radicals, and comparable efficiencies between ZnO NPs and the standard were obtained at higher ZnO concentrations (≥100 µg mL^−1^). In this assay, the IC_50_ value for ZnO@OFE NPs and the standard material were 74.05 µg mL^−1^ (*R*^2^ = 0.793) and 38.37 µg mL^−1^ (*R*^2^ = 0.675), respectively.

#### 3.4.3. Hydrogen Peroxide Scavenging Assay

Although H_2_O_2_ by itself is not particularly reactive, it can occasionally be hazardous to cells because it can trigger the formation of free hydroxyl radicals. Therefore, the elimination of H_2_O_2_ is an extremely critical step, for instance, in the process of protecting food systems. Figure 11c displays an increase in the peroxide scavenging percentage with increase in concentration of ZnO@OFE NPs. Analysis of the inhibition percentages revealed IC_50_ values of 55.26 µg mL^−1^ (*R*^2^ = 0.792) and 20.87 µg mL^−1^ (*R*^2^ = 0.825) for ZnO@OFE NPs and gallic acid, respectively. An antioxidant efficacy of 81% was achieved for 200 µg mL^−1^ of ZnO@OFE NPs, which outperforms that previously reported (~70%) for *Curcuma longa*-assisted ZnO NPs using the same concentration [61].

#### 3.4.4. Superoxide Scavenging Assay

As super oxides are capable of oxidizing both DNA and protein, they posed a threat to the body cells. Figure 11d also depicts the dependence of the scavenging power of ZnO NPs towards superoxide radical on the particles’ concentration. The superoxide scavenging percentage by ZnO@OFE NPs showed an IC_50_ value of 81.37 µg mL^−1^ (*R*^2^ = 0.786) and that of the gallic acid was 48.73 µg mL^−1^ (*R*^2^ = 0.814).

The scavenging mechanism of ZnO is based on its ability to neutralize free radicals and reactive oxygen species (ROS) [62]. The mechanism involves adsorption of the free radicals and ROS on ZnO surface, then electron transfer from ZnO to stabilize the radicals, and the subsequent formation of ZnO-oxide species that can scavenge ROS.

## 4. Conclusions

ZnO NPs were successfully synthesized using a green, simple and eco-friendly approach, in which ethanolic extract of *O. europaea* fruit waste was used as a capping agent and reducing agent. The biosynthesized ZnO@OFE NPs were characterized using a variety of techniques. UV-Vis analysis revealed the high quality of the ZnO@OFE NPs, with a LSPR absorption peak at around 382 nm. The modification of the NPs with the extract capping agent was confirmed by FTIR analysis, where several functional groups from phytochemicals compounds in the extract were revealed. XRD verified that the ZnO@OFE NPs has a pure wurtzite structure. The synthesized ZnO@OFE NPs were spherical with an average particle size of 57 nm. ZnO@OFE NPs exhibited enhanced photocatalytic activity towards MB and MO dyes in the presence of diffused sunlight. The dyes significantly degraded at ZnO@OFE with degradation efficiency increased with time reaching 75 and 87% within only 180 min for MB and MO, respectively. At these NPs, the degradation rate of MO (0.013 min^−1^) is higher than MB (0.008 min^−1^). In addition, the synthesized ZnO@OFE NPs demonstrated significant antioxidant activity against DPPH, OH^−^, peroxide and superoxide free radicals when compared to gallic acid as the standard reference. This study demonstrates the potential of exploiting waste of olive fruit as a raw material for the synthesis of stable ZnO NPs with applications in wastewater treatment, and as a free radical scavenging agent.

## Figures and Tables

**Table 1:** Comparison of the photodegradation parameters of MB and MO at ZnO@OFE.

Dye	Adsorption Efficiency/%	Degradation Efficiency/%	*k* × 10^3^ min^−1^	R^2^
MB	12	75	8	0.991
MO	10	87	13	0.978
4-NP	--	77	2	0.938

**Table 2:** Comparison of the photocatalytic activity of ZnO NPs synthesized using plant extracts and other relevant catalysts from literature.

Catalyst	Preparation Method/ Plant	Dye	Irradiation	Irradiation Time/ min	Catalyst Dose/ g L^−1^	Dye Conc./ mg L^−1^	Degradation Efficiency/%	*k* × 10^3^ min^−1^	Ref.
**ZnO**	Biosynthesis/ *Cassia fistula*	MO	UV (364 nm)	120	1	10	84	--	[49]
**ZnO**	Biosynthesis/ *Syzygium cumini*	MB MO	UV (365 nm)	60 150	2 1.5	1–2	84 88	35 12	[50]
**ZnO**	Biosynthesis/ Camellia sinensis	MB	UV (10 W)	10	1	15	75	--	[51]
**Fe-doped** **ZnO**	Biosynthesis/ wild olive	MO	Sunlight	90	5	10	92	25	[52]
**ZnO**	Sol gel	MB	UV (Hg lamp 365 nm)	120	0.33	10	37	11	[53]
**P25** **Degussa**	--	MB MO	UV lamp	60	1	10	41 43	--	[54]
**ZnO@OFE**	Biosynthesis/sunlight/ *O. europaea*	MB MO	Sunlight	180	1	10	75 87	8 13	This work

## Data Availability

All experimental supporting data and procedures are available within this article.
